# P-1046. Isavuconazonium Utilization in Pediatric Patients at a Free-Standing Children’s Hospital

**DOI:** 10.1093/ofid/ofae631.1236

**Published:** 2025-01-29

**Authors:** Regina Orbach, Jessica Bee, Jamie Legaspi

**Affiliations:** Children's Hospital Los Angeles, Los Angeles, California; Children's Hospital Los Angeles, Los Angeles, California; Children's Hospital Los Angeles, Los Angeles, California

## Abstract

**Background:**

Isavuconazonium sulfate is a newer triazole antifungal used for the treatment of invasive aspergillosis and mucormycosis. In December 2023, the FDA expanded approval for isavuconazonium sulfate (Cresemba) to include pediatric patients 1 year and older. The most common adverse effect requiring monitoring is hepatotoxicity. Routine therapeutic drug monitoring is not recommended for most patients but may be considered if there is a concern for toxicity, therapeutic failure, or impaired drug absorption. With limited published data in pediatric patients, our aim is to evaluate the use of isavuconazonium at Children's Hospital Los Angeles (CHLA) and assess safety and tolerability in our patient population.

**Methods:**

A retrospective chart review was performed to evaluate utilization of isavuconazonium from 01/01/2023 – 12/31/2023 at CHLA. Demographic data, Infectious Diseases consultation, indication for therapy, liver function before and during treatment, and serum drug level results were collected and analyzed.

**Results:**

A total of 54 patient encounters included an isavuconazonium order during the review period. All study patients had a primary diagnosis of leukemia or stem cell transplant (SCT), the most common (45%) being Acute Lymphoblastic Leukemia (ALL). 3 patients had mildly elevated liver function tests (LFTs) at baseline, and 6 total patients developed grade 3 or 4 liver toxicity during therapy. The most common indication for treatment was empiric or definitive treatment of invasive fungal infection, with 15 (28%) and 20 (37%) patients, respectively. 19 patients (35%) received off-label prophylaxis for invasive fungal infection due to contraindications to alternative antifungal agents. 33 of 54 patients had at least one serum trough level measured at steady state. Although a therapeutic target range is not well established, 26 patients (79%) achieved levels within typical expected range of 1-5 mg/L. Of 3 patients with trough level < 1 mg/L, 2 were determined to be due to non-adherent to outpatient therapy.

**Conclusion:**

Based on serum drug levels and LFT results collected for routine therapeutic monitoring, isavuconazonium appears well-tolerated for treatment or prophylaxis of invasive fungal infection in our immunocompromised pediatric population.

**Disclosures:**

**All Authors**: No reported disclosures

